# Bio-Mapping of Microbial Indicators and Pathogen Quantitative Loads in Commercial Broiler Processing Facilities in South America

**DOI:** 10.3390/foods12193600

**Published:** 2023-09-28

**Authors:** David A. Vargas, Gabriela K. Betancourt-Barszcz, Daniela R. Chávez-Velado, Angelica Sánchez, Rossy Bueno López, Marcos X. Sanchez-Plata

**Affiliations:** Department of Animal and Food Sciences, Texas Tech University, Lubbock, TX 79409, USA; andres.vargas@ttu.edu (D.A.V.); gabbetan@ttu.edu (G.K.B.-B.); daniela.r.chavez@ttu.edu (D.R.C.-V.); angelica.sanchez@ttu.edu (A.S.); rossy.bueno@ttu.edu (R.B.L.)

**Keywords:** *Salmonella* enumeration, *Campylobacter* spp. enumeration, poultry bio-mapping, microbial baseline

## Abstract

A bio-mapping study was conducted with the aim of creating a microbiological baseline on indicator organisms and pathogens in commercial broiler processing facilities located in a country in South America. Whole chicken carcass and wing rinses were collected from five stages of the poultry processing line: live receiving (LR), rehanger (R), post-evisceration (PE), post-chilling (PC), and wings (W). Rinses (*n* = 150) were enumerated using the MicroSnap™ system for total viable counts (TVC) and Enterobacteriaceae (EB), while the BAX^®^-System-SalQuant^®^ and BAX^®^-System-CampyQuant™ were used for *Salmonella* and *Campylobacter*, respectively. TVC and EB were significantly different between stages at the processing line (*p* < 0.01). There was a significant reduction from LR to PC for both microbial indicators. TVC and EB counts increased significantly from PC to W. *Salmonella* counts at PC were significantly different from the other stages at the processing line (*p* = 0.03). *Campylobacter* counts were significantly higher than the other stages at PC (*p* < 0.01). The development of bio-mapping baselines with microbial indicators showed consistent reduction up to the post-chilling stage, followed by an increase at the wings sampling location. The quantification of pathogens demonstrates that prevalence analysis as a sole measurement of food safety is not sufficient to evaluate the performance of processing operations and sanitary dressing procedures in commercial processing facilities.

## 1. Introduction

Between the years 1970 and 2005, poultry meat production increased faster than that of beef or pig meat. Although the overall contribution of developing countries to the export volume of poultry products is lower in comparison to that of developed countries, considerable steady growth in the poultry industry has been seen in South America, driven largely by increased consumer demand for higher quality and safer protein sources [1,2]. Consequently, a significant opportunity has emerged for the South American poultry industry to expand its presence in international markets. However, to tap into these global opportunities, it is imperative that their products align with the regulatory standards set by each respective country. These regulatory frameworks are established and enforced by various agencies, such as the United States Department of Agriculture Food Safety and Inspection Service (USDA-FSIS), if the intent is to export their products to the United States [1,3].

Poultry meat is considered one of the most common foods that can cause foodborne illnesses, and studies suggest that it has been associated with approximately 25% of outbreaks caused by foodborne pathogens [4]. Two major target organisms for control in poultry processing facilities are *Salmonella* and *Campylobacter* spp. There is a significant risk of contamination by these pathogens during poultry processing, which can affect the state of finished products [1,5]. Limited information is available concerning foodborne illnesses associated with pathogens in developing countries. Additionally, in many South American countries, limited resources pose a significant obstacle to conducting comprehensive case–control studies that would otherwise help investigate the origins of outbreaks, consequently restricting the reporting of foodborne illness incidents [6,7]. Nevertheless, *Campylobacter* has been associated with 11.3 to 21% of diarrhea episodes in low-income countries [3]. South American countries showed a higher prevalence for *Campylobacter* of almost 100%, which can be linked to an increasing risk of *Campylobacter* infections in humans [3]. In terms of *Salmonella,* studies have shown there is variability between facilities and regions when it comes to *Salmonella* prevalence. The estimated prevalence for *Salmonella* in developing countries was 55.5% [8]. Such high prevalence levels may be due to intestinal tearing during evisceration and cross contamination during scalding, defeathering, and chilling processes [2,9]. 

There is a growing need for official microbial baselines so processors can measure performance and compare data to national and international reference sources. These baselines not only inform risk but promote and reinstate a sense of compliance with the microbial performance standard, where processing facilities have the responsibility to demonstrate the level of control within their food safety systems [1]. For example, the USDA-FSIS implements microbial performance standards based on prevalence (positive or negative) in poultry processing facilities for *Salmonella* [10]. The standard consists of 5 positive results out of 51 samples collected (for whole birds) and eight positive results out of 52 samples collected (for parts) [10]. Processing plants are subsequently grouped according to their performance, utilizing these findings, and allocated to one of three classifications. Category 1 includes facilities that have attained 50% or less of the highest permissible positive percentage in the most recently concluded 52-week rolling period. Category 2 encompasses facilities that satisfy the maximum allowable positive percentage criteria but have results surpassing 50% of the highest allowable positive percentage in the most recently completed 52-week moving window. Category 3 encompasses facilities that have exceeded the maximum allowable positive percentage in the most recently completed 52-week rolling period [11]. Furthermore, the European Union regulation is guided by the National Control and Surveillance Programs (NCP) for *Salmonella*. Although programs may vary between countries and regions, the basis is on the same food safety underlying principle of systematic implementation of preventive and control measures [12]. 

The rising popularity and demand for poultry meat have been accompanied by regulatory advancements in the industry to ultimately ensure the provision of safe meat to consumers. The introduction of Hazard Analysis and Critical Control Point (HACCP) systems constituted the beginning of these advancements to add control measures and improve food safety [5]. For instance, many establishments include antimicrobial interventions such as peracetic acid, chlorine, trisodium phosphate, or cetylpyridinium chloride during their processing to reduce the risk of contamination with pathogens [5,13,14]. Chlorine has been used to facilitate decontamination in washes and chillers during evisceration, but in some cases, its efficacy gets compromised with high organic loads and pH levels above 7.0, which rapidly declines the availability of free chlorine [15,16,17]. Peracetic acid is one of the most common antimicrobials used in processing due to its high efficacy in reducing contamination [13,18,19]. Nevertheless, it has been reported that peracetic acid may potentially cause negative flavor or color changes, resulting in undesirable changes in meat quality [15,18]. The effect of antimicrobial interventions on product quality is still an ongoing and active area of interest for further exploration. 

Given the limited availability of data on microbial indicators and pathogen loads at various stages within poultry processing facilities in Latin American countries, this study aimed to address this knowledge gap. The primary objective was to construct a bio-mapping system that includes indicator organisms and pathogens commonly associated with the poultry industry. This bio-mapping can serve as a valuable tool for establishing microbial baselines related to the evaluation of process changes as a whole system. The ultimate goal was to generate essential information that can facilitate the process of conducting comprehensive risk analyses specific to poultry products within a particular country.

## 2. Materials and Methods

### 2.1. Sample Collection

The study was conducted in 2022 on three commercial processing facilities that account for 75% of the total production of broilers in a South American country, according to data collected during 2021. Whole chicken carcass (1.5–2 kg) and part rinses (1.8 kg) were collected at five different locations throughout the slaughter, evisceration, and de-boning process using 400 mL of buffered peptone water (BPW) (Millipore Sigma, Danvers, MA, USA). Five locations were sampled in the process, including live receiving, where a warm and intact recently identified dead bird on arrival (DOA) was collected as a representation of initial loads entering the facility, re-hanger, post-evisceration; where a whole carcass was sampled before getting into the chiller, after a manual or mechanical evisceration process, post-chiller, and skin-on parts (wings). Sampling consisted of five samples per location, in five different locations for two different days per plant, resulting in a grand total of 150 samples. Locations were selected due to the highest likelihood of contamination or the presence of an intervention step in the process. Rinses were immediately chilled (2–4 °C) and transported to a microbiological laboratory for analysis.

### 2.2. Intervention Parameters

The standard processing conditions for all three commercial processing facilities involved multiple chicken washes with water during the evisceration stage, followed by the use of 1 ppm of residual chlorine in the water exiting the chiller. No other chemical intervention was used throughout the slaughter, evisceration, and de-boning process.

### 2.3. Salmonella Enumeration and Prevalence

After homogenization of the rinses, 30 mL of the rinse was mixed with 30 mL of pre-warmed (42 °C) SalQuant solution (Hygiena, Camarillo, CA, USA) for poultry rinses and immediately incubated at 42 °C for 6 h for recovery. Then, the BAX^®^-System-SalQuant^®^ (Hygiena, Camarillo, CA, USA) methodology was followed for the enumeration of *Salmonella*. Then, samples were incubated for 18 h at 42 °C for enrichment. Samples that were not positive for enumeration using BAX^®^-System-SalQuant^®^ (Hygiena, Camarillo, CA, USA) were tested for prevalence analysis using the BAX^®^ System RT-*Salmonella* Assay (Hygiena, Camarillo, CA, USA) [10,20].

### 2.4. Campylobacter Enumeration and Prevalence

After rinses were homogenized, 30 mL of the rinse was combined with 30 mL of pre-warmed (42 °C) CampyQuant solution (Hygiena, Camarillo, CA, USA) for poultry rinses and immediately incubated at 42 °C for 20 h for recovery under microaerophilic conditions (6 to 16% O_2_ and 2 to 10% CO_2_) using BD GasPak EZ Campy Sachets (Becton Dickinson and Company, Franklin Lakes, NJ, USA) in BD GasPak EZ Container Systems (Becton Dickinson and Company, Franklin Lakes, NJ, USA). After recovery, the BAX^®^-System-CampyQuant™ (Hygiena, Camarillo, CA, USA) methodology was followed for the enumeration of *Campylobacter*. Samples were then incubated for 28 h at 42 °C for enrichment. Samples that were not positive for enumeration using BAX^®^-System-CampyQuant™ (Hygiena, Camarillo, CA, USA) were tested for prevalence analysis using the BAX^®^ System RT-*Campylobacter* Assay (Hygiena, Camarillo, CA, USA).

### 2.5. Microbial Indicators Enumeration

Upon arrival, rinses were homogenized by hand and serially diluted in 9 mL BPW tubes (Millipore, Danvers, MA, USA) for enumeration of microbial indicators. The MicroSnap™ system (Hygiena, Camarillo, CA, USA) was used for the enumeration of total viable counts (TVC) and Enterobacteriaceae (EB). Quantification consists of a two-step process where 1 mL of the rinse was added to the MicroSnap ™ Enrichment Device Step #1 (Hygiena, Camarillo, CA, USA) and mixed with the enrichment broth provided by the kit. Snaps were incubated at 30 ± 0.5 °C for 7 h ± 10 min for TVC and 37 ± 0.5 °C for 6 to 8 h following protocol guidelines for enumeration range. After incubation, enriched sample from the MicroSnap ™ Enrichment Device Step #1 (Hygiena, Camarillo, CA, USA) was transferred to MicroSnap ™ Detection Device Step #2 (Hygiena, Camarillo, CA, USA) and mixed with the detection liquid provided by the kit. Snaps were immediately inserted into an Ensure Touch luminometer (Hygiena, Camarillo, CA, USA) and results were provided in relative light units (RLU) that were finally converted into CFU/mL. This study also includes a validation component that outlines the utilization of MicroSnap ™ technology for the quantification of indicator microorganisms.

### 2.6. MicroSnap Validation

A cocktail of five distinct strains of *Salmonella* (*Salmonella enterica* subsp. *enterica* ser. Enteritidis (ATCC 31194), *Salmonella enterica* subsp. *enterica* ser. Typhimurium (BAA 712), *Salmonella enterica* subsp. *enterica* ser. Infantis (BAA 1675), *Salmonella enterica* subsp. *enterica* ser. Senftenberg (ATCC 43845), *Salmonella enterica* subsp. *enterica* ser. Newport (ATCC 6962)) was used for this validation. Microorganisms were streaked onto brain heart infusion (BHI) agar plates (Millipore Sigma, Danvers, MA, USA) and incubated at 37 °C for 24 h. A single well-isolated colony of each serotype was suspended in 5 mL of sterile phosphate-buffered saline water (PBS) (Sigma-Aldrich, St. Louis, MO, USA) until a calibrated concentration of approximately 1~2 × 10^8^ CFU/mL was attained using the Thermo Scientific™ Sensititre™ Nephelometer (Thermo Fisher, Waltham, MA, USA) set to 0.5 McFarland turbidity. Individual suspensions were then combined in a 15 mL conical tube to create the final cocktail. After serial dilution, eight tubes containing a concentration ranging from 1 × 10^1^ to 1 × 10^8^ were used for enumeration. Enumeration was performed using: (1) Direct plating using drop plating and micro dilution on Tryptic Soy Agar (TSA) plates (Millipore Sigma, Danvers, MA, USA), (2) MicroSnap™ system (Hygiena, Camarillo, CA, USA), (3) APC 3M ™ Petrifilm ™ (3M, Saint Paul, MN, USA), and (4) TEMPO^®^ System (BioMérieux, Paris, France). Four repetitions were conducted throughout the whole study.

(1) Direct plating. For each independent tube, 10 and 100 µL for drop and spread plating, respectively, were plated on TSA (Millipore Sigma, Danvers, MA, USA) according to each dilution. Plates were incubated for 16 h at 37 °C.
(2) MicroSnap ™ system. For each independent tube, the same protocol described in Section 2.5 for total viable counts was followed.
(3) APC 3M ™ Petrifilm ™: For APC in Petrifilms, the Association of Official Agricultural Chemists 990.12 (AOAC) official method was used. Aerobic plate count petrifilms were incubated for 48 ± 3 h at 35 ± 1 °C.
(4) TEMPO^®^ System: Cards were incubated for 22–28 h at 35 ± 1 °C following AOAC 121,204 for aerobic counts (AC). After incubation, the cards were inserted into the TEMPO^®^ reader (BioMérieux, Paris, France), and counts were calculated by the system.

### 2.7. Statistical Analysis

The analysis of all collected data was conducted using R (Version 4.1.3) statistical analysis software. The analysis aimed to assess variations in microbial loads across different processing line locations, specifically live receiving, rehanger, post-evisceration, post-chiller, and wings. To facilitate data visualization and analysis, the counts were transformed into Log CFU/mL for aerobic counts and Enterobacteriaceae (EB). Conversely, for *Salmonella* and *Campylobacter* spp., counts were reported as Log CFU/sample (Log CFU/rinse). For each microorganism, a one-way ANOVA analysis was initially performed, comparing the counts observed at each of the five locations. Subsequently, a pairwise comparison *t*-test adjusted with the Tukey–Kramer method was carried out. In cases where the parametric assumptions required for ANOVA were not met, the Kruskal–Wallis test was employed as a non-parametric alternative followed by a pairwise comparison using the Wilcoxon’s test, with adjustments made using the Benjamin and Hochberg method. All significant differences were assessed with a *p*-value below 0.05.

For MicroSnap ™ validation, data were analyzed using R (Version 4.1.3) statistical analysis software to evaluate the relation between counts obtained from MicroSnap ™, 3M™ Petrifilm™, TEMPO^®^ System, and direct plating. A linear model was calculated where log_10_ counts from the MicroSnap ™ method were considered as the independent variable, and log_10_ counts from direct plating, 3M™ Petrifilm™, and TEMPO^®^ System were considered as the dependent variables.

## 3. Results

**Figure 1:**
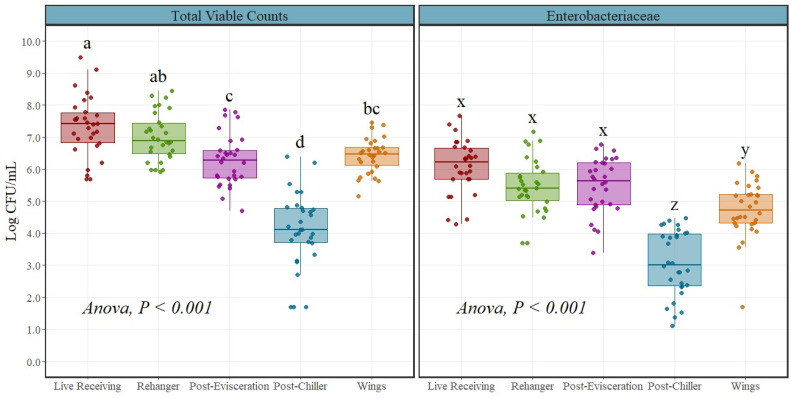
Enterobacteriaceae and total viable counts (Log CFU/mL) at different locations throughout the poultry processing line (*n* = 30 rinses per location/organism). In every boxplot, the median is depicted by the horizontal line intersecting the box, while the lower and upper quartiles are denoted by the bottom and top of the box, respectively. The vertical line extending upwards represents 1.5 times the interquartile range, and the vertical line extending downwards signifies 1.5 times the lower interquartile range. ^(a–d)^ Boxes within aerobic counts labeled with distinct letters exhibit statistically significant differences as determined using ANOVA analysis, followed by pairwise comparisons using t-test adjusted Tukey at a significance level of *p* < 0.05. (x–z) Boxes within Enterobacteriaceae counts labeled with distinct letters exhibit statistically significant differences as determined using ANOVA analysis, followed by pairwise comparisons using *t*-test adjusted Tukey at a significance level of *p* < 0.05. The points represent the actual data points.

Enterobacteriaceae and total viable counts (Figure 1) were reported in Log CFU/mL, while *Salmonella* and *Campylobacter* spp. were transformed to Log CFU/sample, equivalent to Log CFU/400 mL. *Salmonella* and *Campylobacter* spp. counts, when reported as Log CFU/mL, resulted in negative values, making the analysis and visualization hard to interpret. The BAX^®^-System-SalQuant^®^ and BAX^®^-System-CampyQuant^™^ methodology used for the quantification of both pathogens have a limit of quantification (LOQ) of 1 CFU/mL or 400 CFU/sample after the recovery phase; then, if the sample was negative for quantification, a prevalence test is performed with a limit of detection (LOD) of 0.0025 CFU/mL or 1 CFU/sample. In order to be able to perform an analysis that includes both the quantification and prevalence results, some adjustments were made to the data (Table 1).

BAX^®^-System-SalQuant^®^ and BAX^®^-System-CampyQuant ™ counts can be extrapolated below the LOQ as counts are obtained from a regression equation provided by methodology, the reason why a new LOQ was established as 1% of the real LOQ (0.01 CFU/mL or 0.6 Log CFU/sample) [10,20,21,22]. Counts below 0.6 Log CFU/sample were reported as 50% of the new LOQ (0.3 Log CFU/sample), as well as samples that were not quantifiable but observed positive for prevalence analysis. Finally, samples that were not quantifiable nor detected were reported as 0 Log CFU/sample. A summary of the adjustments used for the data analysis can be observed in Table 1.

### 3.1. Indicator Microorganisms

For total viable counts, the incoming microbial level was measured from DOA at the live receiving area with an average count of 7.34 Log CFU/mL. After stunning, killing, scalding, and feather removal, counts were measured at the rehanger area with an average count of 7.01 Log CFU/mL. Subsequently, after the evisceration process, counts were significantly reduced (*p* < 0.001) to an average value of 6.28 Log CFU/mL and finally reduced to microbial levels around 4.10 Log CFU/mL after finishing the whole slaughtering process in the production line (post-chiller). An overall trend was observed with higher levels at the live receiving area with a continuous decrease until post-chiller and a final significant increase (*p* < 0.001) in parts (Wings) reaching levels around 6.43 Log CFU/mL (Figure 1 and Table 2).

A similar trend was observed for Enterobacteriaceae counts, with an average incoming microbial level of 6.08 Log CFU/mL followed by a slight reduction at the rehanger step with an average value of 5.46 Log CFU/mL. No statistical difference was observed between the live receiving area, rehanger, and post-evisceration steps (*p* > 0.05), as counts at the post-evisceration step were similar to counts at the rehanger area (5.49 Log CFU/mL). A significant reduction (*p* < 0.001) was observed at post-chiller with average microbial counts of 3.07 Log CFU/mL, followed by a significant increase (*p* < 0.001) in parts reaching levels around 4.74 Log CFU/mL (Figure 1 and Table 2).

### 3.2. Salmonella Prevalence and Enumeration

In this microbial baseline study, *Salmonella* counts were notably low when assessed per mL. Consequently, all data were converted to Log CFU/sample, which is equivalent to Log CFU/400 mL. Additionally, reported averages at each sampling stage take into consideration all observations collected during the study, including observations that were negative for *Salmonella* presence making the average value go towards zero due to prevalence levels on each sampling stage. 

**Figure 2:**
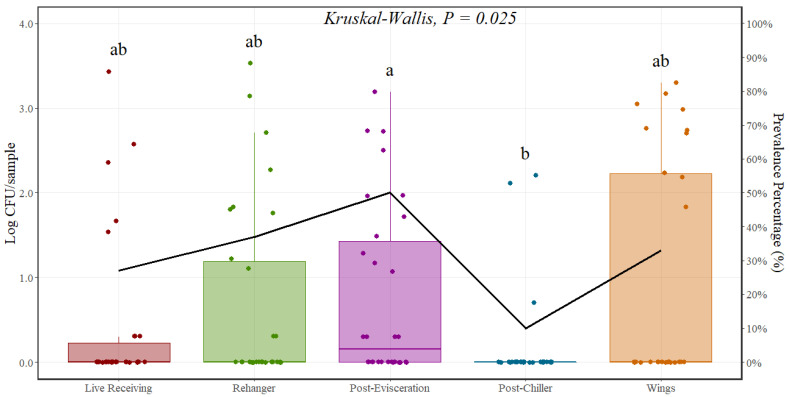
*Salmonella* prevalence (%) and counts (Log CFU/sample) at different locations throughout the poultry processing line (*n* = 30 rinses per location). In every boxplot, the median is depicted by the horizontal line intersecting the box, while the lower and upper quartiles are denoted by the bottom and top of the box, respectively. The vertical line extending upwards represents 1.5 times the interquartile range, and the vertical line extending downwards signifies 1.5 times the lower interquartile range. (a–b) Boxes labeled with distinct letters exhibit statistically significant differences as determined by Kruskal–Wallis analysis, followed by pairwise comparisons using Wilcoxon’s test adjusted with the Benjamin and Hochberg method, at a significance level of *p* < 0.05. The points represent the actual data points.

The average incoming *Salmonella* count measured at the live receiving area was 0.42 Log CFU/sample. These counts were obtained from DOA prior to any chemical intervention representing the actual incoming level to the processing plants. Counts at the rehanger area and post-evisceration area were 0.67 and 0.77 Log CFU/sample, respectively. A significant reduction was observed (*p* < 0.001) at the post-chiller area when compared with the post-evisceration step, with an average decrease of around 0.60 Log CFU/sample. *Salmonella* levels at parts were 0.90 Log CFU/sample (Figure 2 and Table 2). In addition to enumeration, *Salmonella* prevalence results are presented in Table 3.

### 3.3. Campylobacter Prevalence and Enumeration

**Figure 3:**
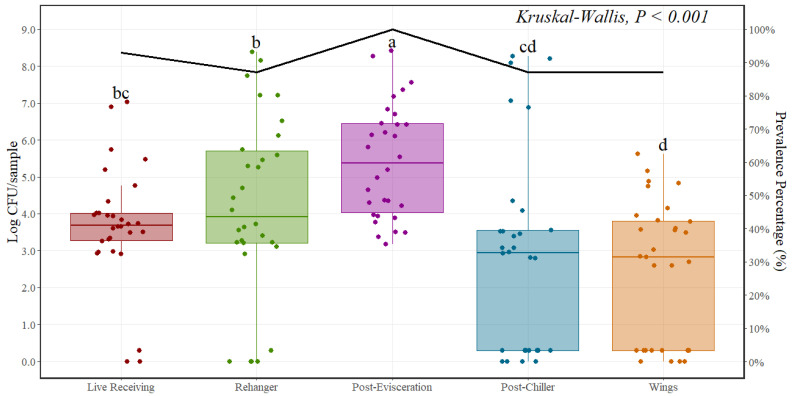
*Campylobacter* spp. prevalence (%) and counts (Log CFU/sample) at different locations throughout the poultry processing line (*n* = 30 rinses per location). In every boxplot, the median is depicted by the horizontal line intersecting the box, while the lower and upper quartiles are denoted by the bottom and top of the box, respectively. The vertical line extending upwards represents 1.5 times the interquartile range, and the vertical line extending downwards signifies 1.5 times the lower interquartile range. (a–d) Boxes labeled with distinct letters exhibit statistically significant differences as determined by Kruskal–Wallis analysis, followed by pairwise comparisons using Wilcoxon’s test adjusted with the Benjamin and Hochberg method, at a significance level of *p* < 0.05. The points represent the actual data points.

*Campylobacter* counts were also transformed to Log CFU/sample, and the average value included observations that were negative for *Campylobacter* presence. This was less affected than *Salmonella* averages because the prevalence of *Campylobacter* at all stages was higher. The average incoming level prior to any chemical intervention was 3.68 Log CFU/sample. Contrary to indicator microorganism results, counts increased at the rehanger area to 4.18 Log CFU/sample and significantly increased (*p* < 0.001) at post-evisceration with an average of around 5.42 Log CFU/sample. A significant reduction was observed at post-chilling and parts (*p* < 0.001) with *Campylobacter* counts around 2.81 and 2.46 Log CFU/sample (Figure 3). In addition to enumeration, *Campylobacter* prevalence results are presented in Table 3. 

### 3.4. MicroSnap Validation

**Figure 4:**
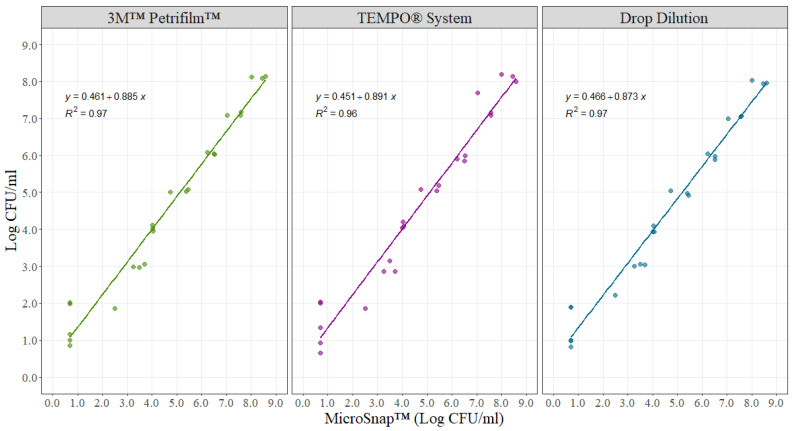
Visualization of the linear relationship between bacterial counts in Drop Dilution, 3M™ Petrifilm™, and TEMPO^®^ System compared to MicroSnap ™. (*n* = 32 per method).

For the MicroSnap ™ validation experiment, counts were log_10_ transformed and then analyzed. The slope in the linear model signifies the rate of change in microbial counts when transitioning from the MicroSnap™ method to one of the alternative methods (3M™ Petrifilm™, TEMPO^®^, or drop dilution) for every 1 unit increase. Ideally, a slope close to one indicates that for each 1 Log CFU/mL increase observed with an alternative method, there is a corresponding 1 Log CFU/mL increase with the MicroSnap™ method. Furthermore, the intercept represents the measurement obtained with any of the alternative methods when the MicroSnap™ method registers zero, indicating the similarity in the overall magnitude readings when comparing the two methods. The slopes for the three alternative methods, when compared to the MicroSnap™ method, were 0.885, 0.891, and 0.873 for 3M™ Petrifilm™, TEMPO^®^, and drop dilution, respectively (Figure 4). Correspondingly, the intercept value was 0.461 (3M™ Petrifilm™), 0.451 (TEMPO^®^), and 0.466 (drop dilution). All three intercepts were statistically different from zero (*p* = 0.019 for 3M™ Petrifilm™), (*p* = 0.046 for TEMPO^®^), and (*p* = 0.008 for drop dilution). 

## 4. Discussion

Microbial indicator bio-mapping is a novel approach for processors to identify the ongoing changes in indicator bacteria within a specific system because it provides an overview of how effectively their process maintains low microbial levels [23]. Conducting a comprehensive enumeration throughout the process allows facilities to identify critical steps that enhance the safety of the final product. In addition, it helps identify steps with minimal microbial impact that could potentially be modified or removed because their influence on the system is limited [23]. Moreover, when microbial baselines are combined with bio-mappings, a solid database can be generated to make comparisons between studies, change regulations, or modify intrinsic factors of the process, such as antimicrobial levels, new processing schemes, or speed lines [10]. 

For both indicator organisms, a similar trend was observed in microbial levels with a minimal reduction from the live receiving area to the post-evisceration step, followed by an important reduction at post-chiller and then a significant increase in parts (wings). The increase in microbial loads on parts has been observed in multiple studies, and it has been associated with the extensive manipulation intrinsic to the process (cutting, processing, and packaging) that increases the potential for cross-contamination [10,21,24]. However, the existing sampling method can significantly influence the results because the contact surface during parts rinsing is larger compared to whole chicken carcass rinsing, which affects the final counts.

Enumeration of total viable counts and Enterobacteriaceae resulted in reductions of 3.24 and 3.01 Log CFU/mL, respectively, during the slaughtering and evisceration process. In other bio-mapping studies, reductions in aerobic counts of 6.17 and 5.72 Log CFU/mL and Enterobacteriaceae counts of 5.13 and 5.11 Log CFU/mL were reported from live receiving area to post-chiller under normal chemical interventions and reduced chemical interventions in a plant located in the United States, respectively [10]. Discrepancies in the results are due to differences of the process itself, poultry processing facilities in the United States usually use a series of washes and chemical interventions to control the microbial levels in chicken carcasses at multiple steps of the process to ensure food safety [10,15,25]. Some of the most important chemicals used are acidified sodium chlorite (ASC), cetylpyridinium chloride (CPC), chlorine dioxide, and peroxyacetic acid (PAA), which are the most widely used and effective [26]. Conversely, as described in the methodology, all plants included in this study either have only one chemical intervention at the chiller stage or none at all, depending on the destination of the final product. Some products exported to Europe prohibit the use of antimicrobial chemical interventions during slaughter or processing to prevent concealing unhygienic practices and preventing microflora resistance on the product’s surface [27]. Another important difference can be described during the evisceration process, mechanical vs. manual evisceration. A study compared two poultry processing facilities (manual evisceration and mechanical evisceration, respectively) were the greatest incidence of contamination with the pathogen on the manual evisceration plant was observed in the product while in the mechanical evisceration plant was observed in non-food contact surfaces [28]. The effect of manual evisceration can be clearly seen by looking at pathogens numbers with an increase in the Log CFU/sample at the post-evisceration process. The likelihood of cross contamination during manual evisceration is greater when compared with mechanical evisceration. This resembles the importance of correct and constant training to plant workers to minimize the occurrence of carcass contamination with intestinal and extra-intestinal source of pathogens. 

Most *Salmonella* and *Campylobacter* research studies on poultry processing facilities focused mainly on prevalence analysis, whereas in this current study, the quantification of pathogens using the polymerase chain reaction (PCR) technique allowed the creation of a unique and comprehensive baseline for the country to provide data for risk analysis and decision making as well as provide information about medium to small size poultry processing plants in developing countries [5,29]. The importance of pathogen quantification can be clearly shown in results obtained for *Campylobacter*, the overall prevalence throughout the whole process is barely different from stage to stage, ranging from 85% to 100%, suggesting that the process itself is not making any control at keeping this pathogen out, but when looking at the loads of the pathogen, it can be shown that there is a reduction at the counts specially after the chiller. The interpretation of the results can be completely different if only looking at prevalence values, the reason why the inclusion of pathogen enumeration is crucial to see the real effect of a specific process or intervention. 

By looking at *Salmonella* results and comparing them with other studies, the results were not as expected, as normally higher prevalence and loads were supposed to be observed at the first stages of the slaughter process with reductions throughout the whole line [10]. At the live receiving area, the background flora on incoming levels of *Salmonella* may cause an overwhelming/inhibitory effect during the DNA extraction and later replication step during the PCR analysis, causing difficulties in the methodology. Normally, to compensate for this possible effect, a recovery phase of 6 h with selective media is performed to avoid difficulties during the molecular analysis. *Salmonella* prevalence result at the post-chiller step was 10%, which is close to the maximum allowable percent positive level established by the United States Department of Agriculture (9.8%) [11]. According to the regulation, three categories are defined depending on the prevalence level of *Salmonella* on samples taken after the chilling step. Category 1 or consistent process control are establishments that achieve 50% or less of the maximum allowable percent positive during a 52-week moving window over the last 13 weeks [11]. Category 2 or variable process control are establishments at or below the maximum allowable percent positive for all completed 52-week moving windows [11]. Category 3 or high variable process control are establishments with greater than the maximum allowable percent positive during the same gap of time [11]. The discussion about levels and categories established by the government is outside the scope of this paper, but the inclusion of quantification as part of the categorization of poultry processing facilities is a topic that should be considered, as the risk of illness is highly dependent on the microbial load, especially on *Salmonella.*

The use of the MicroSnap™ technology for the quantification of indicator microorganisms was used and tested during this study due to the easiness and convenience offered by the methodology to obtain results in places where well-equipped laboratory facilities may not exist. The flexibility of MicroSnap™ is tied to practical equipment with a flexible and easy-to-use format that facilitates its use as a “mobile lab”. In order to support the use of this methodology for this project, a correlation experiment between MicroSnap™ and other commonly used methodologies (3M™ Petrifilm™, TEMPO^®^, or drop dilution) was performed. For instance, when comparing MicroSnap™ to 3M™ Petrifilm™, the estimated slope obtained during regression analysis was 0.885 Log CFU/mL, suggesting that for 1 Log CFU/mL increase in counts using MicroSnap™, a 0.885 Log CFU/mL increase in counts will be obtained using the 3M™ Petrifilm™ methodology. The 3M™ Petrifilm™ 95% confidence interval of the slope did not contain the value of 1, which means that the slope was statistically different from 1, suggesting that there are minimal differences between both methodologies for the quantification of microorganisms (Table 4). When comparing MicroSnap™ to drop dilution, the slope obtained was 0.873 Log CFU/mL, which means that for 1 Log CFU/mL increase in counts using MicroSnap™, a 0.873 Log CFU/mL increase in counts is obtained using the more traditional and laborious methodology of drop dilution. In the case of the TEMPO^®^ system, the estimated slope was 0.891, so for a 1 Log CFU/mL increase in counts using MicroSnap™, a 0.891 increase in counts is obtained from the TEMPO^®^ method. Both drop dilution and TEMPO^®^ slope 95% confidence intervals did not contain the value of 1, indicating that there are minimal differences when comparing MicroSnap™ to drop dilution and MicroSnap™ to TEMPO^®^, respectively (Table 4). 

The MicroSnap™ technology is an enumeration method that uses a rapid bioluminogenic test reaction that generates light when certain enzymes that are specific or characteristic of viable bacteria react with the substrate to produce light [30]. The light-generating signal is then quantified with a Hygiena™ luminometer that translates RLU to CFU following the Baranyi-Roberts model of bacteria growth in foods [31]. The results are obtained in 6 to 8 h depending on the microorganisms tested, giving the advantage of knowing microbial loads on same-day samples in order to make decisions based on data [32]. Based on an intercept-based assessment, the intercepts of the three other methods were significantly different from zero at a significance level of 0.05, indicating that there are minimal differences when comparing MicroSnap™ to three common methods for enumerating microorganisms. This information serves as additional supporting evidence for the use of MicroSnap™ in this study. 

## 5. Conclusions

The microbiological profile obtained in this study will not only provide a microbial baseline for the country but also a comparative guideline for other countries in South America with small to medium size poultry processing facilities. Access to this information can help the country inform the decision-making process for continuous food safety improvement in their food safety system in addition to conducting risk analysis of their poultry products. Microbiological baseline studies should be conducted in all countries in order to have historical data to compare for improvements in food safety systems as well as a way to measure the performance of a specific facility or country. Furthermore, the findings of this study suggest that the quantification of pathogens demonstrates that prevalence analysis as a sole measurement of food safety is not sufficient to evaluate the performance of processing operations and sanitary dressing procedures in commercial processing facilities.

## Figures and Tables

**Table 1:** Observed and adjusted parameters established for *Salmonella* and *Campylobacter* spp. quantification and prevalence analysis for statistical analysis and graphical representation.

Observed SalQuant or CampyQuant Result (Log CFU/Sample)	Observed *Salmonella* or *Campylobacter* spp. Prevalence Result	Adjusted SalQuant or CampyQuant Result (Log CFU/Sample)	Adjusted *Salmonella* or *Campylobacter* spp. Prevalence Result
No Result	Negative	0	Negative
No Result	Positive	0.3	Positive
Less than 0.6	NA ^1^	0.3	Positive
More or equal than 0.6	NA	Observed SalQuant or CampyQuant result	Positive

^1^ NA: not applicable, as prevalence analysis is not necessary for samples quantified by SalQuant or CampyQuant.

**Table 2:** Summary table of indicator and pathogen counts for all sampling locations throughout the chicken processing plants (*n* = 30 rinses/sampling location).

Sampling Location	Microorganism			
	Total Viable Counts (LogCFU/mL ± SE ^1^)	Enterobacteriaceae (LogCFU/mL ± SE)	*Salmonella* (LogCFU/Sample ^2^ ± SE)	*Campylobacter* spp. (LogCFU/Sample ± SE)
Live Receiving	7.34 ± 0.17 ^a^	6.08 ± 0.15 ^a^	0.42 ± 0.16 ^ab^	3.68 ± 0.29 ^bc^
Rehanger	7.01 ± 0.14 ^ab^	5.46 ± 0.16 ^a^	0.67 ± 0.20 ^ab^	4.18 ± 0.45 ^b^
Post-Evisceration	6.28 ± 0.15 ^c^	5.49 ± 0.16 ^a^	0.77 ± 0.19 ^a^	5.42 ± 0.28 ^a^
Post-Chiller	4.10 ± 0.21 ^d^	3.07 ± 0.19 ^c^	0.17 ± 0.10 ^b^	2.81 ± 0.49 ^cd^
Wings	6.43 ± 0.17 ^bc^	4.74 ± 0.16 ^b^	0.90 ± 0.24 ^ab^	2.46 ± 0.35 ^d^
*p*-value	<0.001	<0.001	0.025	<0.001

^1^ Standard error of the mean. ^2^ LogCFU/sample is equivalent to LogCFU/400 mL. ^(a–d)^ For total viable counts and enterobacteriaceae, values with different letters are significantly different according to ANOVA analysis followed by pairwise comparison *t*-test at *p* < 0.05. For *Salmonella* and *Campylobacter* spp., values with different letters are significantly different according to Kruskal–Wallis analysis followed by pairwise comparison Wilcoxon’s test at *p* < 0.05.

**Table 3:** Prevalence percentage of *Salmonella* and *Campylobacter* spp. for all sampling locations throughout the chicken processing plants.

Sampling Location	Microorganism	
	*Salmonella* (%)	*Campylobacter* spp. (%)
Live Receiving	26.67% (8/30)	93.33% (28/30)
Rehanger	36.67% (11/30)	86.67% (26/30)
Post-Evisceration	50.00% (15/30)	100.00% (30/30)
Post-Chiller	10.00% (3/30)	86.67% (26/30)
Wings	33.33% (10/30)	86.67% (26/30)

**Table 4:** Summary table of linear model using the least square regression method predicting the bacterial counts on Drop Dilution, 3M™ Petrifilm™, and TEMPO^®^ System when compared with MicroSnap ™.

Enumeration Method	Coefficient	Estimate	Standard Error	*p*-Value	95% Confidence Intervals	
					Lower (2.5%)	Upper (97.5%)
Drop Dilution	Intercept	0.466	0.161	0.008	0.132	0.801
	Slope	0.873	0.030	<0.001	0.810	0.937
3M ™ Petrifilm ™	Intercept	0.461	0.181	0.019	0.085	0.837
	Slope	0.885	0.034	<0.001	0.814	0.956
TEMPO^®^ System	Intercept	0.451	0.213	0.046	0.009	0.893
	Slope	0.891	0.040	<0.001	0.807	0.974

## Data Availability

Data is available on request from the corresponding author. The data are not publicly available due to privacy from the beef processing partner that allowed the project to be conducted within their beef processing environment.
